# Analysis of Chinese Consumers’ Nutrition Facts Table Use Behavior Based on Knowledge-Attitude-Practice Model

**DOI:** 10.3390/ijerph182212247

**Published:** 2021-11-22

**Authors:** Zeying Huang, Haijun Li, Jiazhang Huang

**Affiliations:** 1 Institute of Food and Nutrition Development, Ministry of Agriculture and Rural Affairs, Beijing 100081, China; huangzeying@caas.cn; 2 School of Information &Intelligence Engineering, University of Sanya, Sanya 572022, China; haijunli1968@163.com

**Keywords:** nutrition facts table, nutrition labeling, knowledge-attitude-practice model, Chinese residents, structural equation model

## Abstract

The nutrition facts table is a nutrition labeling tool designed to help consumers understand the nutritional content of food and make informed food choices. However, the utilization rate of the nutrition facts table has been low in China since its introduction in 2013. This study employed structural equation modeling to investigate consumers’ knowledge, attitudes, and practices related to the nutrition facts table by using an online survey of 930 valid samples from 31 provinces in China based on the knowledge, attitude, practice (KAP) model. The analysis revealed that most respondents had a positive attitude, but not a good level of knowledge or practice in their use of the labeling. Respondents’ knowledge about the nutrition facts table directly affected their attitude, through which it could then affect their practice, whereas knowledge played an indirect role in nutrition labeling use. The results confirmed that the KAP model is suitable for explaining consumers’ use of nutrition labels in China, and that the first step to promoting labeling usage is to enrich consumers’ knowledge on the nutrition facts table.

## 1. Introduction

The nutrition facts table is a nutrition labeling tool designed to inform consumers of the nutritional properties of a food [1] and meet consumers’ requirement for accurate, standardized and comprehensible information on the content of food items in order to make healthy choices [2]. The nutrition facts table is a standardized statement or listing of the nutrient content of a food, and it is widely applied in countries such as the United States [3], Britain [4], Canada [5], and Australia [6].

**Figure 1:**
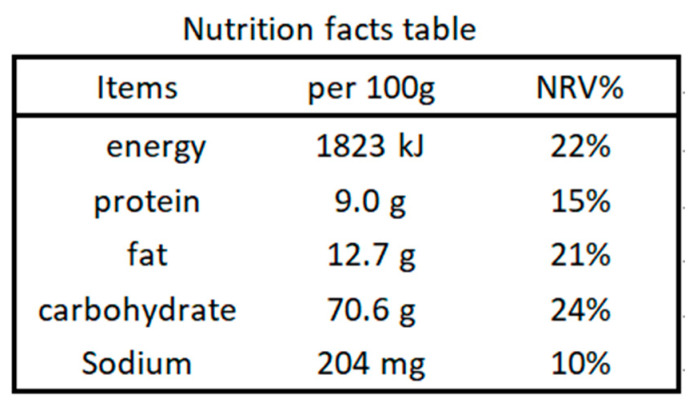
A sketch map of a nutrition facts table. Source: Chinese Nutrition Society [12].

Obesity is a growing problem in China and has been linked to a variety of adverse health outcomes (e.g., diabetes, heart disease, and certain cancers). The Report on Nutrition and Chronic Diseases in China (2020) illustrated that over 50% of adults were overweight or obese in 2019, with an increase of at least 8% compared with the year 2012 [7]. To improve residents’ diet quality, since 2013 China has implemented the nutrition facts table, a major and mandatory nutrition labeling that provides the energy value and the amount of protein, fat, carbohydrates, and sodium, as well as the percentages of nutrient reference values (NRV) per 100 g (mL) of a prepackaged food (see Figure 1) [8]. However, the nutrition facts table is rarely used by Chinese consumers when shopping for food [9,10]. To increase the use of nutrition labels by consumers, China has conducted various dietary education programs such as National Nutrition Week activities, which have been held every year in the third week of May for the promotion of nutrition knowledge from 2015 to the present, but to date these have had limited effects [11].

The knowledge, attitude, practice (KAP) model is a theory used to interpret an individual’s healthy behavior [13]. Up to now, it has been widely used in the field of infectious diseases (e.g., schistosomiasis, dengue fever) prevention [14,15] and chronic diseases control (e.g., diabetes, hypertension) [16,17]. Nutrition labeling use by consumers is a dietary self-management behavior, and the KAP model therefore serves as an appropriate framework to explore the relationship between knowledge, attitude, and practice towards the nutrition facts table. Some studies have used the KAP model and monofactor analysis to assess knowledge, attitude, and practice regarding information on food labels among university students in Malaysia [18,19], medical and non-medical sciences students in Iran [20], and consumers in Thailand [21]. Others sought to identify the effects of health education interventions on employees’ knowledge, attitude, and practice towards using food labels in Sirjan University [22]. However, few studies have investigated the causal relationship among knowledge, attitude, and practice. Furthermore, research on the nutrition facts table in China has focused on residents’ understanding and use of the label [9,10], but without KAP surveys. Therefore, this study attempts to fill this void by using the KAP model to investigate interactions between knowledge, attitude, and practice related to nutrition labeling among Chinese consumers, and test whether the KAP model is suitable for analysis of nutrition facts table use in China.

## 2. Hypotheses of the KAP Model

**Figure 2:**
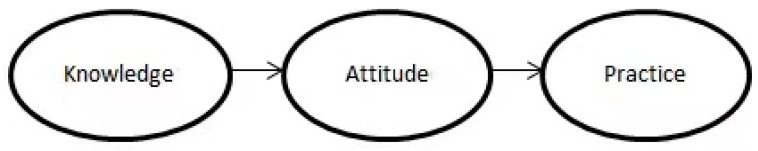
The KAP model. Source: Wang et al. [25].

As is showed in Figure 2, the KAP model proposes that individual practice (P) is a process consisting of knowledge (K) and attitude (A). Only by acquiring knowledge can one take a positive attitude to changing behavior [23]. The KAP model was initially applied to family planning surveys in the 1970s [24] and was later used to explain an individual’s behavior in the public health management field.

In our study, knowledge refers to understanding of or information about the nutrition facts table acquired through experience or education, while attitude is a feeling or opinion about the nutrition facts table, and practice refers to action on or application of the labeling. According to the KAP model, knowledge about the nutrition facts table is expected to have a positive and indirect effect on practice (behavior) by changing attitude. Consumers’ attitude towards the nutrition facts table interacts with their knowledge, thereby leading to usage. Thus, the following hypotheses are proposed:

**Hypothesis** **1** **(H1).** *Consumers who have higher-level knowledge about the nutrition facts table are more likely to have a positive attitude towards it.*

**Hypothesis** **2** **(H2).** *Consumers who have a more positive attitude towards the nutrition facts table are more likely to use nutrition information.*

**Hypothesis** **3** **(H3).** *Consumers with a higher level of knowledge about the nutrition facts table are more likely to use nutrition labeling information.*

## 3. Methods and Materials

### 3.1. Measures and Collection of Data

According to the KAP model, an individual’s knowledge, attitude, and practice towards the nutrition facts table can be measured by related questions listed in Table 1. To assess three latent variables in Table 1, scale items were obtained based on information labeled on China’s nutrition facts tables and its function. According to prior studies [19,26], all items were evaluated on a 5-point Likert scale, ranging from 1 ‘strongly disagree’ to 5 ‘strongly agree’. For this study, a self-administered questionnaire was developed, containing 16 questions (Tables S1–S4), after it was pre-tested on 80 residents at two randomly selected districts in Beijing on 21 October 2020.

The Wenjuanxing platform (https://www.wjx.cn accessed on 10 November 2020), as one of the leading companies specializing in online questionnaire data collection in China, has a sample pool of 2.6 million potential respondents reasonably distributed by gender, age, and region. Paid survey data collection services are provided by Wenjuanxing platform for sending questionnaires to target samples and ensuring the validity of questionnaire information.

From 10 November to 28 December, 2020, the Wenjuanxing platform was commissioned to collect 930 valid survey samples nationwide from the sample pool. Firstly, a stratified sampling approach was employed to randomly select 45 individuals from each of China’s 31 provinces/autonomous regions to complete online questionnaire surveys, and then cross-checking was conducted to eliminate invalid questionnaires due to lack of information and implausible answers. Finally, a total of 930 valid samples (i.e., 30 samples × 31 regions) were generated for analysis.

### 3.2. Methods

Propositions that link exogenous variables with endogenous variables were analyzed by structural equation modeling (SEM), which is reliable in examining the relationships between different constructs (i.e., differences among groups of latent variables), and provides accurate and meaningful results [27]. Compared with other techniques, it allows us to create several indicator variables (i.e., observable variables) per construct, which does not require the split analysis method and yields valid and clear inferences [28]. Therefore, the results of the relationships among variables are reliable and neutral [29]. Additionally, SEM has the capability to scrutinize complicated associations and a variety of hypotheses by simultaneously incorporating mean structures and group estimation [30]. Hence, the hypotheses proposed above were tested by structural equation modeling. Specifically, all data analyses were performed in two stages. First, this study employed SPSS version 25.0 (IBM Corporation: Armonk, NY, USA) to conduct evaluation of measured items’ stability and consistency by reliability analysis. Second, the assessment of the goodness-of-fit of the structural equation model and hypotheses testing were analyzed by moment structure through AMOS version 21.0 (IBM Corporation: Armonk, NY, USA).

## 4. Results

The sample is representative of the Chinese population in terms of socio-demographic characteristics. Table 2 summarizes the demographic profile of our sample. Overall, respondents were predominantly male (56.77%), aged between 18 and 44 years (31.61%), with a high school level education (31.29%), and had an after-tax middle income from 10,000 to 50,000 Yuan (27.31%).

Table 3 presents the characteristics of respondents’ knowledge, attitude, and practice towards the nutrition facts table. With regard to the concept and function of nutrient reference values (NRV), most respondents (about 42%) had little knowledge. Regarding energy and main nutrients, fewer people had knowledge about the NRV of energy and sodium. For attitude towards the nutrition facts table, more than half of respondents believed that the label could be used to compare nutrients among similar foods, choose healthy food, and understand nutrients of food. As to use of the nutrition facts table, less than 50% of respondents practiced it, and fewer respondents used the nutrition facts table as a food purchase reference than to compare nutrients among similar foods.

### 4.1. Analysis of Discriminant Validity

Table 4 illustrates the statistically significant correlations among constructs through Pearson’s correlation test. The square root of average variance extracted (AVE) was to be greater than its correlation with other constructs after assessment of discriminant validity with AVE.

### 4.2. Goodness-of-Fit Test

Exploratory factor analysis (EFA) was employed to examine factor structure and reduce the number of items. The suitability of factor analysis was determined with the Kaiser–Meyer–Olkin (KMO) test and Bartlett’s test of sphericity (BTS). The BTS was significant (χ2 = 1082.60, *p* < 0.05) and the condition of EFA was met. The KMO value was above 0.7, which suggests the items are suitable for factor analysis as recommended by Kaiser [31]. The consistency of all constructs’ items was tested by composite reliability (CR). AVE and item loadings were used to examine the convergent validity for the purpose of evaluating the level of association between items [32]. The findings revealed that the AVE values exceeded 0.50 for all constructs and suggested that the latent constructs retained a minimum of 50% variance.

A reliability analysis was conducted to test the reliability of the samples. It was advised that the reliability coefficient must be greater than 0.70 [33]. Table 5 indicates that the value of CR and Cronbach’s α exceeded 0.70 for all constructs and the data were valid and reliable.

### 4.3. Valuation of Structural Equation and Hypothesis Testing

**Figure 3:**
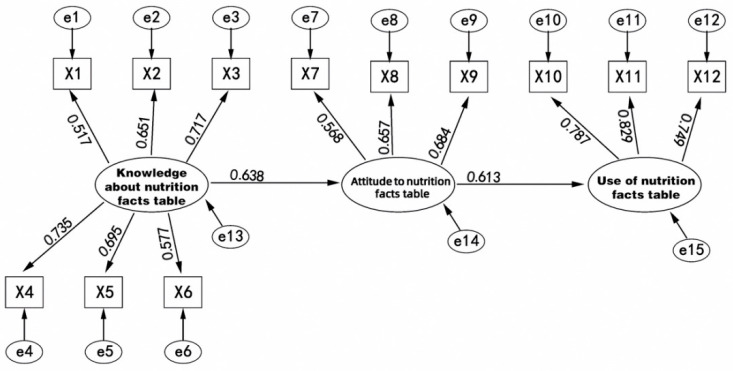
Results of structural equation modeling. Comparative fit index = 0.916; goodness-of-fit index = 0.933; root mean square error of approximation = 0.072; degrees of freedom = 52; chi-square = 374.583. X1–X12 are the scale items codes and e1–e15 are statistical errors of 3 variables and 12 scale items.

The hypothesized relationships were analyzed after the validity and reliability of the measures were attained. Figure 3 shows the path analysis of the structural model. The goodness-of-fit indices for the structural model were calculated (see Table 6). Each of the fitting index values (SCS = 2.583, CFI = 0.916, IFI = 0.916, GFI = 0.933, AGFI = 0.901, RMSEA = 0.072, NNFI = 0.993, NFI = 0.904) outperformed the respective threshold value, signifying that the data fit the structural model satisfactorily [34,35].

As Table 7 showed, Hypothesis 1 is supported by evidence that the path coefficient of H1 is 0.638 (*p* < 0.001), indicating that consumers’ knowledge level on the nutrition facts table was positively and significantly associated with their attitude. Hypothesis 2 (0.613, *p* < 0.001) is supported and suggests that consumers’ attitude significantly and positively influences their use of the nutrition facts table. Two similar path coefficients indicated that the influence of an individual’s knowledge about the nutrition facts table on his or her attitude has a comparable magnitude as the influence of attitude on practice. In addition, Hypothesis 3 is rejected because an individual’s knowledge about the nutrition facts table is not statistically correlated with his or her use of it, suggesting that the level of knowledge amongst Chinese consumers has no relationship with their behavior.

## 5. Discussion

This is the first study to report on nutrition facts table usage among consumers through the KAP model in China. The findings verify the value and function of individuals’ knowledge, attitude, and practice in nutrition labeling use, and also provide deep insight into linkages between consumers’ knowledge, attitude, and practice regarding the nutrition facts table. The key strength of this study is its adoption of the KAP model to analyze Chinese consumers’ nutrition facts table usage behavior, which was innovatively divided into knowledge, attitude, and practice. Regarding methodological strength, structural equation modeling is superior to multiple regression modeling. Its application was helpful to analyze the direct effects of KAP and to reveal these relationships. However, the present study has a few limitations. First of all, more rigorous survey questions are required. For example, participants were likely to make inaccurate responses, since the concept and function of information on the nutrition facts table are two different elements of knowledge. Furthermore, respondents’ knowledge of nutrition facts table needs to be objectively measured, rather than through the use of self-reported questions. Finally, in context of the whole Chinese population of 1.4 billion people, our sample size was not large enough and hardly ensured that the findings above could be replicated or supported within behavioral studies; hence, a larger sample is worth studying in future studies.

### 5.1. Association between Knowledge and Attitude of the Nutrition Facts Table

The significant relationship between knowledge and attitude regarding nutrition labeling showed that respondents could form a more positive attitude towards the nutrition facts table if they were knowledgeable about the labeling. Evelyn et al., [19] found positive correlations between knowledge and attitudes among food labeling, while Rimpeekool et al., [21] reported similar results among consumers. This correlation reflects that once an individual accepts or acquires the knowledge of nutrition facts tables, he or she is likely to establish belief towards using nutrition labeling by thinking, and then forms a positive attitude [21]. Most surveyed consumers had little and incomplete knowledge of the nutrition facts table, but more than half of respondents believed that the nutrition facts table played a role in consumption guidance. The main reason may be that the promotion of the nutrition facts table in China focuses more on cultivating consumers’ positive attitude towards the labeling, but less on education of relevant knowledge. For example, the *Dietary Guidelines for Chinese Residents* places more emphasis on labeling promotion than detailed explanations.

### 5.2. Association between Attitude and Practice of the Nutrition Facts Table

Rimpeekool et al. [21] reported a positive and significant relationship between consumers’ attitudes and practices regarding food labeling among consumers in Thailand. Our study showed such a relation, with those with more positive attitudes towards the nutrition facts table associated with a high likelihood of using nutrition labeling, so attitude was identified as an important factor in influencing labeling adoption practices. It is possible that an individual’s attitude is a psychological response to convincing himself or herself that food labeling could be useful, and that individuals’ labeling use behavior then becomes gradually formed by their attitude [18]. Hence, in this study, Chinese respondents’ attitude towards the nutrition facts table served as a crucial mediator between the corresponding knowledge and practice, which was consistent with the KAP theory.

### 5.3. Association between Knowledge and Practice of the Nutrition Facts Table

Data of this study indicated that respondents’ knowledge could not translate directly into nutrition facts table practice. This is in line with the findings of Grunert & Wills [36]. They argued that individual’s knowledge was an indirect motivator to influence his or her nutrition labeling use. One of main reasons given by Nurliyana et al., [18] was that it is a gradual process from acquisition of knowledge to behavior change. Specifically, those with higher knowledge level appear to be more able to interpret the information provided on nutrition labels [18] but the behavior of the label use could not ultimately be formed without belief about function of the labeling. Hence, our findings confirmed that only by attitude could consumers’ knowledge impact their labeling use, and also reflected that abundant knowledge is the basis for changing the behavior of nutrition facts table use. However, the fact that Chinese consumers have a low level of knowledge in this area is one of the major constraints to labeling use. Therefore, the synchronous development of KAP regarding nutrition labeling should be paid attention to, and this study highlights the importance of enhancing Chinese consumers’ knowledge level of nutrition labeling.

## 6. Conclusions and Recommendations

Utilizing data gathered from an online survey across China, this study revealed that more than half of respondents had a positive attitude towards the nutrition facts table, but without sufficient relevant knowledge or a high utilization rate of the labeling. Another key finding was that the use of the KAP model is suitable for analyzing Chinese consumers’ usage behavior of the nutrition facts table. Consumers’ knowledge was found to be linked to their use of the nutrition facts table due to an indirect association, while consumers’ attitude towards the nutrition facts table, which was found to be positively affected by their knowledge, showed a significantly positive impact on their nutrition labeling use behavior. In order to cultivate a healthy lifestyle using the nutrition facts table at the point of purchase, the following policy recommendations are offered. First, more public education programs such as printed graphic propaganda, special lectures, and learning websites should be designed and implemented in order to improve consumers’ relevant knowledge and consequently increase the use of nutrition labeling. Specifically, explanations of the nutrition facts table knowledge need to be included in the *Dietary Guidelines for Chinese Residents*, and the nutrition labeling-related knowledge should be disseminated as much as possible in National Nutrition Week activities. Second, the concept and function of NRV and major nutrients on the nutrition facts table, especially sodium, need to be communicated in an easy-to-understand manner, and the explanation of the terms of NRV should be displayed on the prepackaged food, ensuring better comprehension by consumers. Last but not least, this study calls for effective supervision and inspection to be implemented to ensure the accuracy of the information labeled on the nutrition facts table, in order to enhance consumers’ confidence in the function of nutrition labeling.

## Figures and Tables

**Table 1:** Latent variables and scale items.

Latent Variables	Scale Items
	I know the concept and function of nutrient reference values (NRV) from the nutrition facts table.
	I know the concept and function of energy information from the nutrition facts table.
Knowledge about the nutrition facts table	I know the concept and function of carbohydrate information from the nutrition facts table.
	I know the concept and function of fat information from the nutrition facts table.
	I know the concept and function of protein information from the nutrition facts table.
	I know the concept and function of sodium information from the nutrition facts table.
	I believe the nutrition facts table could be used to compare nutrients among similar foods.
Attitude towards the nutrition facts table	I believe the nutrition facts table could be used to choose healthy food.
	I believe the nutrition facts table could be used to understand nutrients in food.
	I read the nutrition facts table when food shopping.
Use of the nutrition facts table	I use the nutrition facts table as a food purchase reference.
	I use the nutrition facts table to compare nutrients among similar foods.

**Table 2:** Samples’ sociodemographic characteristics.

Attributes	Sorts	Numbers	%
Sex	Male	528	56.77
	Female	402	43.23
Age	Below 18 years old	168	18.06
	18–44	294	31.61
	45–59	275	29.57
	60 years old and above	193	20.76
Education level	Primary school and below	127	13.66
	Junior high school	283	30.43
	High school	291	31.29
	College/Bachelor	198	21.29
	Postgraduate or above	31	3.33
Annual household income (after tax)	Below 10,000 Yuan	117	12.58
	10,000–50,000 Yuan	254	27.31
	50,001–100,000 Yuan	246	26.45
	100,001–150,000 Yuan	180	19.35
	150,001–200,000 Yuan	83	8.92
	Above 200,000 Yuan	50	5.38

One US dollar equaled 6.524 Chinese Yuan and one euro equaled 7.960 Chinese Yuan from 10 November to 28 December, 2020.

**Table 3:** Description of variables and summary statistics.

Variables	Scale Items	Strongly Disagree		Disagree		Neither Agree Nor Disagree		Agree		Strongly Agree	
		*N*	Percentage%	*N*	Percentage%	*N*	Percentage%	*N*	Percentage%	*N*	Percentage%
Knowledge about the nutrition facts table	I know the concept and function of nutrient reference values (NRV) from the nutrition facts table.	105	11.29	388	41.72	313	33.66	113	12.15	11	1.18
	I know the concept and function of energy information from the nutrition facts table.	60	6.45	310	33.33	375	40.32	160	17.20	25	2.69
	I know the concept and function of carbohydrate information from the nutrition facts table.	62	6.67	208	22.37	336	36.13	277	29.78	47	5.05
	I know the concept and function of fat information from the nutrition facts table.	43	4.62	179	19.25	310	33.33	323	34.73	75	8.06
	I know the concept and function of protein information from the nutrition facts table.	43	4.62	159	17.10	305	32.80	340	36.56	83	8.92
	I know the concept and function of sodium information from the nutrition facts table.	112	12.04	266	28.60	337	36.24	163	17.53	52	5.59
Attitude towards the nutrition facts table	I believe the nutrition facts table could be used to compare nutrients among similar foods.	28	3.01	147	15.81	289	31.08	404	43.44	62	6.67
	I believe the nutrition facts table could be used to choose healthy food.	21	2.26	83	8.92	261	28.06	431	46.34	134	14.41
	I believe the nutrition facts table could be used to understand nutrients of food.	29	3.12	72	7.74	245	26.34	411	44.19	173	18.60
Use of the nutrition facts table	I read the nutrition facts table when food shopping.	45	4.84	272	29.25	258	27.74	220	23.66	135	14.52
	I use the nutrition facts table as a food purchase reference.	79	8.49	275	29.57	274	29.46	223	23.98	79	8.49
	I use the nutrition facts table to compare nutrients among similar foods.	90	9.68	231	24.84	294	31.61	233	25.05	82	8.82

**Table 4:** Factor correlations and discriminant validity.

Factors	Knowledge about the Nutrition Facts Table	Attitude to the Nutrition Facts Table	Use of the Nutrition Facts Table
Knowledge about the nutrition facts table	0.734		
Attitude to the nutrition facts table	0.638 **	0.710	
Use of the nutrition facts table	0.391 **	0.613 ***	0.728

Values in brackets were the square-root AVE. Significance levels (*** *p* < 0.001, ** *p* < 0.01). Matrix diagonals represent the square root of AVE while the other entries represent the squared correlations.

**Table 5:** Factor loadings and convergent validity results.

Variables	Scale Items Code	Scale Items	Standard Loadings	AVE	Composite Reliability	Cronbach’ s Alpha
Knowledge about the nutrition facts table	X1	I know the concept and function of nutrient reference values (NRV) from the nutrition facts table.	0.517	0.539	0.833	0.813
	X2	I know the concept and function of energy information from the nutrition facts table.	0.651			
	X3	I know the concept and function of carbohydrate information from the nutrition facts table.	0.717			
	X4	I know the concept and function of fat information from the nutrition facts table.	0.735			
	X5	I know the concept and function of protein information from the nutrition facts table.	0.695			
	X6	I know the concept and function of sodium information from the nutrition facts table.	0.577			
Attitude to the nutrition facts table	X7	I believe the nutrition facts table could be used to compare nutrients among similar foods.	0.568	0.504	0.757	0.798
	X8	I believe the nutrition facts table could be used to choose healthy food.	0.657			
	X9	I believe the nutrition facts table could be used to understand nutrients of food.	0.684			
Use of the nutrition facts table	X10	I read the nutrition facts table when food shopping.	0.787	0.530	0.719	0.830
	X11	I use the nutrition facts table as a food purchase reference.	0.829			
	X12	I use the nutrition facts table to compare nutrients among similar foods.	0.749			

Rotation technique: Promax; extraction technique: maximum likelihood; total variance elucidated: 63.03%; Bartlett’s test of sphericity: χ2 =1082.60; Kaiser–Meyer–Olkin measure of sampling adequacy: 0.736 (*p* < 0.001); AVE: average variance extracted.

**Table 6:** Structural equation modeling fitting.

Goodness-of-Fit Indices	Fitting Index Values	Fitting
Standard Chi–Square (SCS)	2.583	<3, good
Comparative Fit Index (CFI)	0.916	>0.9, good
Incremental Fit Index (IFI)	0.916	>0.9, good
Goodness-of-fit Index (GFI)	0.933	>0.9, good
Adjusted Goodness-of-fit Index (AGFI)	0.901	>0.9, good
Root Mean Square Error of Approximation (RMSEA)	0.072	<0.08, good
Non-Normalizing Fitting Index (NNFI)	0.993	>0.9, good
Norm Fitting Index (NFI)	0.904	>0.9, good

**Table 7:** Test results of the hypothesis.

Hypothesized Paths	Normalized Path Coefficient	*T* Value	Accepted
H1: Knowledge about the nutrition facts table→ Attitude to the nutrition facts table	0.638 ***	2.743	Yes
H2: Attitude to the nutrition facts table→ Use of the nutrition facts table	0.613 ***	13.460	Yes
H3: Knowledge about the nutrition facts table→ Use of the nutrition facts table	0	0	No

Notes: levels of statistical significance (*** *p* < 0.00)

## Data Availability

Data is contained within the article.

## Supplementary Materials

The following are available online at https://www.mdpi.com/article/10.3390/ijerph182212247/s1, Table S1: Knowledge about nutrition facts table, Table S2: Attitude to nutrition facts table, Table S3: Use of nutrition facts table, Table S4: Demographic characteristics.
