# Supplementary material for: Analysis of Chinese Consumers’ Nutrition Facts Table Use Behavior Based on Knowledge-Attitude-Practice Model
Source: Int J Environ Res Public Health. 2021 Nov 22;18(22):12247. doi: 10.3390/ijerph182212247 (PMC8625815; doi:10.3390/ijerph182212247)
Supplement: Supplementary file 1 [file ijerph-18-12247-s001.zip › ijerph-1390965-supplementary.pdf]

Supplementary materials: The questionnaire.

Table S1. Knowledge about nutrition facts table.

Knowledge about nutrition facts	**strongly disagree**	**disagree**	**neither agree nor disagree**	**agree**	**strongly agree**
I know the concept and function of nutrient reference values (NRV) from the nutrition facts table.					
I know the concept and function of energy information from the nutrition facts table.					
I know the concept and function of carbohydrate information from the nutrition facts table					
I know the concept and function of fat information from the nutrition facts table.					
I know the concept and function of protein information from the nutrition facts table.					
I know the concept and function of sodium information from the nutrition facts table.					

Table S2. Attitude to nutrition facts table.

**Attitude to nutrition facts**	**strongly disagree**	**disagree**	**neither agree nor disagree**	**agree**	**strongly agree**
I believe the nutrition facts table could be used to compare nutrients among similar foods.					
I believe the nutrition facts table could be used to choose healthy food.					
I believe the nutrition facts table could be used to understand nutrients in food.					

Table S3. Use of nutrition facts table.

**Use of nutrition facts**	**strongly disagree**	**disagree**	**neither agree nor disagree**	**agree**	**strongly agree**
I read the nutrition facts table when food shopping.					
I use the nutrition facts table as a food purchase reference.					
I use the nutrition facts table to compare nutrients among similar foods.					

Table S4. Demographic characteristics table.

Demographic characteristics	
Your gender	A. Male<br>B. Female
Your age	A. Under 18 years old<br>B. 18-44 years old<br>C. 45-59 years old<br>D. 60 years old and above
Your education level	A. Primary school and below<br>B. Junior high school<br>C. High school<br>D. College/Bachelor<br>E. Postgraduate or above
Your individual annual income on average (Yu-an)	A. Below 10,000 Yuan<br>B.10,000–50,000 Yuan<br>C. 50,0001–100,000 Yuan<br>D. 100,001–150,000 Yuan<br>E. 150,0001–200,000 Yuan<br>F. Above 200,000 Yuan
